# Occurrence of ‘soft flesh’ condition induced by *Kudoa thyrsites* parasite in the Iberian European sardine stock

**DOI:** 10.1007/s00436-024-08436-2

**Published:** 2024-12-18

**Authors:** Lucilla Giulietti, Gema Hernandez-Milian, Paolo Cipriani, Miguel Bao, Hui-Shan Tung, Carmen Hernández, Arne Levsen

**Affiliations:** 1 Institute of Marine Research (IMR), Nordnesgaten 50, NO-5005 Bergen, Norway; 2 Oceanographic Centre of Vigo, IEO-CSIC, Vigo, Spain; 3 Department of Public Health and Infectious Diseases, Section of Parasitology, Sapienza University of Rome, Rome, Italy; 4 Oceanographic Centre of Santander, IEO-CSIC, Santander, Spain

**Keywords:** ‘Soft flesh’, Quality reducing parasite, *Kudoa thyrsites*, European sardine, Iberian sardine stock, Spanish fishery

## Abstract

European sardine *Sardina pilchardus* is a commercially valuable coastal pelagic fish species. Spain is one of the largest sardine suppliers in Europe and the Iberian stock is of particular significance. *Kudoa* parasites are known to infect sardines causing the so-called ‘soft flesh’ condition; however, data on the occurrence of ‘soft flesh’ in this sardine stock are limited. This study investigates the occurrence of *Kudoa*-induced ‘soft flesh’ in the Iberian sardine stock caught in 2023 off the northern Spanish Atlantic coast (Division 8.c). Five hundred specimens were examined for ‘soft flesh’ by manual texture testing and visual inspection 48 h post-catch using standardized procedures. ‘Soft flesh’ was detected in 5.4% (27/500) of the sardines. Microscopic examination of muscle samples revealed the presence of *Kudoa thyrsites*–like myxospores in all ‘soft flesh’–affected fish, which based on SSU rDNA gene sequence analysis was identified as *K*. *thyrsites*. The unsightly appearance of infected fillets represents a substantial food quality issue for the Iberian sardine stock that could reduce marketability and consumer confidence in both local and international markets. This is particularly relevant since larger Iberian sardines, which are highly appreciated by consumers, appear to be the most affected.

## Introduction

European sardine *Sardina pilchardus* (Walbaum 1792) is a commercially valuable coastal pelagic fish species (Neves et al. 2021), widely distributed in the Eastern Atlantic Ocean from Senegal to the North Sea and throughout most of the Mediterranean Sea (Parrish et al. 1989; Jemaa et al. 2015; Silva et al. 2015). Five distinct sardine stocks have been identified in the Atlantic (FAO 2023; ICES 2023). The northern European stock is distributed in the Celtic Seas (ICES divisions 7a, b, c, f, g, j, k) and the English Channel (ICES divisions 7d, e, h), the central European stock is located in the Bay of Biscay (ICES divisions 8a, b, d), and southern European stock, commonly referred to as the Iberian stock, extends across the Cantabrian Sea, Portuguese waters, and the Gulf of Cádiz (ICES divisions 8.c and 9.a) (ICES 2023). In waters along the West African coast, the Fishery Committee for the Eastern Central Atlantic (CECAF) identifies two main stocks: the Northern African Stock which is distributed from Morocco to Cape Blanc (Mauritania) (FAO 34.1 zones A + B), and southern African stock which ranges from Mauritania to Cape Verde in Senegal (FAO 34.1 zone C) (FAO 2023).

Spain is one of the largest sardine suppliers in Europe. In European Atlantic waters, Spanish purse-seiner vessels primarily target the Iberian sardine stock. Total annual landings of this stock were approximately 16,000 in 2022 and 14,000 tonnes in 2021, marking a remarkable increase from the previous 6 years, during which landings remained below 10,000 tonnes (Silva et al. 2015; ICES 2023). Sardines are an important traditional meal in northern Spain, where they are prepared in various ways including grilling, frying, and canning (Menezes et al. 1990; Molina-Fernández et al. 2015). Due to their high nutritional composition (FAO 2008), sardines are also highly valued in global markets, particularly as canned products (Menezes et al. 1990; Molina-Fernández et al. 2015). Anecdotal instances of flesh softening in the Iberian sardine stock from the northern Spain have been reported by both consumers and fishermen. This condition is believed to be caused by mechanical stress during fishing operations and handling, such as dense onboard storage (personal observation, Miguel Bao). However, factors such as suboptimal storage temperature or bacterial and parasitic infections can also contribute to softening and gaping in the fillets, ultimately affecting quality and marketability of the product (Cheng et al. 2014; Bolin et al. 2021).

At least 15 myxosporean *Kudoa* spp. parasite (Cnidaria, Myxozoa) species are known to significantly impact fish product quality and marketability by inducing myoliquefaction, commonly referred to as ‘soft flesh’ (or ‘jelly flesh’, ‘milky flesh’), characterized by enzymatic degradation of the flesh in post-harvest fish (Moran et al. 1999; Funk et al. 2008; Levsen 2015). Freshly caught fish infected with myoliquefactive *Kudoa* parasites may initially appear as premium products without no signs of spoilage. However, in heavily infected fish (high density), ‘soft flesh’ can emerge 6–48 h post-mortem (St-Hilaire et al. 1997a; Giulietti et al. 2022a), or even after several days or weeks post-mortem in initially frozen and subsequently thawed fish (personal observation, Arne Levsen). This presents a risk as infected fish prone to develop ‘soft flesh’ may enter the market, potentially undermining consumer confidence and causing financial detriment to the fisheries sector (Moran et al. 1999; Levsen 2015; Giulietti et al. 2022a). Cases of flesh softening have been previously documented in sardine from the Iberian Peninsula. The first observations of ‘soft flesh’ in sardines from the Iberian stock were recorded in Portuguese waters (ICES division 9.a.CN) in the 1990s, leading to substantial economic impacts on the regional canning industry (Menezes et al. 1990; Gilman and Eiras 1998). However, research addressing this quality issue in the Iberian sardine stock, especially in northern Spain, remains limited. Previous studies often lacked comprehensive quantitative data on the occurrence of ‘soft flesh’ or did not provide molecular identification of the causative parasite species (Menezes et al. 1990; Gilman and Eiras 1998; Cruz et al. 2011; Iglesias et al. 2022), thus limiting our understanding of this condition in the Iberian sardine stock and related economic and management implications.

This study aimed to investigate the occurrence of *Kudoa*-induced ‘soft flesh’ in the Iberian sardine stock from off the northern Spanish Atlantic coast (ICES division 8.c), and to identify the causative species using molecular methods.

## Materials and methods

### Biological sampling

A total of 500 European sardine individuals were sampled in April 2023 off the northern Spanish Atlantic coast (Cantabrian Sea) (ICES division 8.c) during the research cruise PELACUS0423. Fish were caught using pelagic trawls at 10–200 m on the continental shelf and upper slope off northern Spain, approximately spanning between 43° 45.81′ N 7° 13.77′ W, off Burriana, and 43° 28.29′ N 1° 48.03′ W, off Hondarribia, near the border with France. Onboard, fish were identified to species level, measured (total length, TL; mm), weighed (total weight, TW; g), and sexed. Sex could not be assessed in immature individuals, and thus those were classified as undetermined. If the catch was too big, a randomly selected subsample of fish was taken. Fulton`s condition factor (Fulton’s K) was calculated (K = W(g) × 10^5^/L (mm)^3^) for each fish individual as a proxy of the fish host fitness (Nash et al. 2006). The age of the fish inspected for *Kudoa*-induced ‘soft flesh’ could not be directly determined. Therefore, the fish age was estimated using an age-length key developed from sardines sampled during the same survey as part of national programs under the EU Data Collection Framework. The experimental design did not involve handling live animals, and procedures were conducted in compliance with relevant EU legislation, including EU Directive 2010/63/EU on the protection of animals used for scientific purposes.

### ‘Soft flesh’ examination and parasite species identification

Fish were kept at approximately 10 °C onboard and examined for signs of post-mortem muscle liquefaction 48 h after capture, following the guidelines by Giulietti et al. (2022a, b, 2024), which indicate that ‘soft flesh’ tends to appear in *Kudoa*-infected fish between 6 and 48 h after death when stored at approximately 10 °C. The examination of the fish flesh involved both manual muscle texture testing and visual inspection of the muscle appearance to determine whether the myomere structure remained intact, as described by Levsen et al. (2008). To confirm the occurrence of *Kudoa* spp., two vials (1.5 mL) were filled with the liquefied muscle tissue (approximately 1 cm^3^) from fish scored as soft. Samples were stored at − 20 °C and sent frozen to the Institute of Marine Research in Bergen, Norway, for further analyses.

For microscopic analysis, approximately a quarter (1/4) of the collected muscle tissue was minced using a scalpel and prepared on glass slides moistened with saline water (St-Hilaire et al. 1997b). The slides were then examined under a brightfield microscope (up to 400 × magnification) for myxospores. If spores resembling those of *Kudoa* were observed, a preliminary identification was made based on their morphology, including overall shape and appearance of the spores (Giulietti et al. 2024). Images of spores were taken at 1000 × magnification.

The soft muscle samples were analyzed for *Kudoa* spp. infections by PCR. DNA was extracted from 40 mg of the liquefied muscle tissue using DNeasy® Blood and Tissue Kit (Qiagen) and following the manufacturer’s protocol. The DNA concentration was determined using Quibit™ (Thermo Fisher Scientific, USA). The small subunit ribosomal RNA (SSU rRNA) gene sequences of *Kudoa* spp. were amplified using the primers Ksp18SF (5′-GGA TAA CTG TGG TAA ATC TAG AGC- 3′) and Ksp18SR (5′-GAG CAA TTA TTA CAA GGC TCA RTC- 3′) in accordance with the protocol of Giulietti et al. (2020). PCR products were purified and sequenced by Eurofins (Cologne, Germany). The sequences were assembled using ChromasPro 2.1.5 (Technelysium Pty Ltd., Tewantin, Australia) and aligned with ClustalX 2.0 (Larkin et al. 2007), and their identities were verified through BLAST searches (www.ncbi.nlm.nih.gov/BLAST). New sequences were deposited in GenBank with accession number PQ164243.

### Statistical analysis

The occurrence of ‘soft flesh’ was expressed as the proportion (%) of ‘soft flesh’–affected fish of the total number of fish inspected by manual texture testing and visual inspection, and subsequently confirmed as infected by molecular analysis (Levsen et al. 2008; Giulietti et al. 2022a). Fish TL was used as primary descriptor of fish size in all statistical analyses, due to its correlation with TW. Data normality and homogeneity of variance were assessed using Shapiro–Wilk and Levene’s tests, respectively. Differences in host biometric parameters such as TW, TL, and Fulton’s K between unaffected and ‘soft flesh’–affected fish were tested using the Mann–Whitney *U* test (nonparametric test). To determine any possible effect of sex, the occurrence of ‘soft flesh’ in males and females was compared using Fisher’s exact tests. All statistical analyses were conducted with Statistica® 13.5.0.17 (TIBCO Software Inc., CA, USA).

## Results

**Fig. 1:**
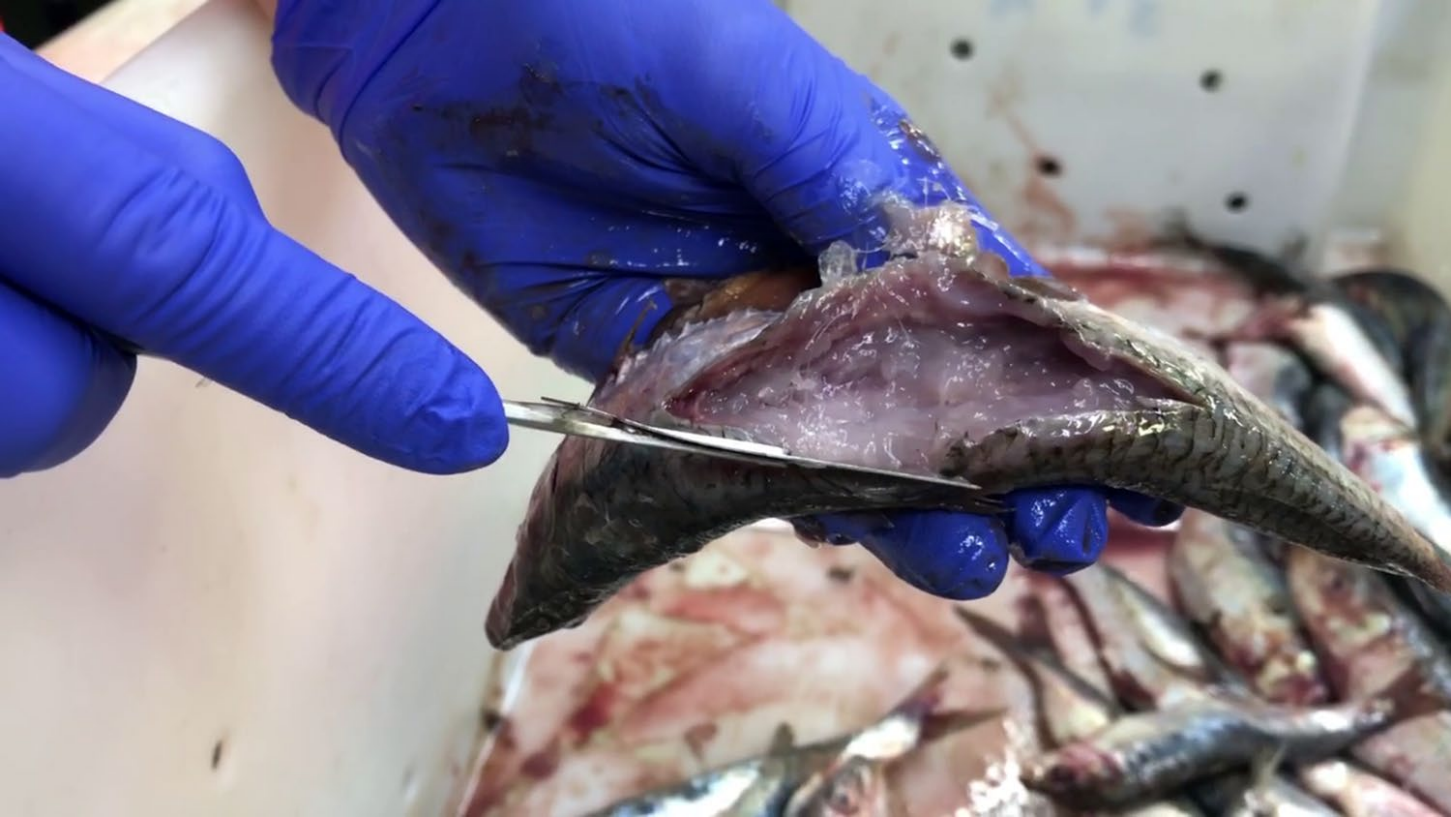
Illustration of *Kudoa*-induced ‘soft flesh’ condition in Iberian sardines from off the northern Spanish Atlantic coast (Cantabrian Sea) (ICES division 8.c.)

**Fig. 2:**
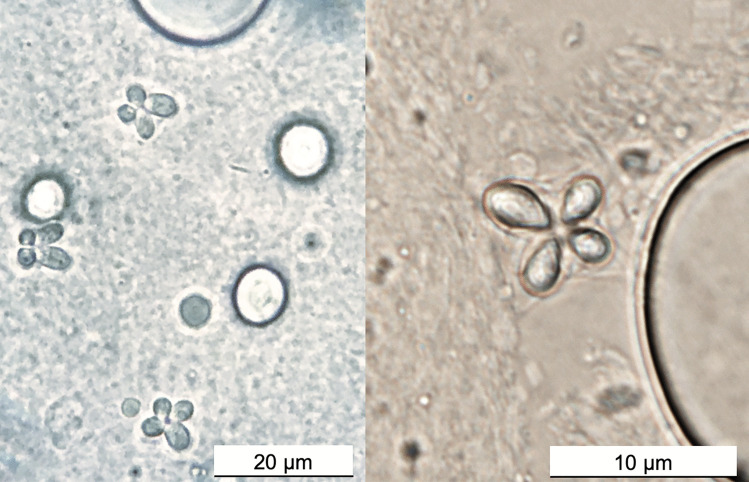
Spores of *K. thyrsites*–like morphotype from Iberian sardine

‘Soft flesh’ was detected in 5.4% of the European sardines sampled (27/500) (Table 1). Fillet exhibiting this condition showed unusually soft and liquefied tissue, with varying degrees of degradation among fish individuals. Some individuals showed a complete breakdown of the segmental myomere structure (Fig. 1). Microscopic examination of the liquefied muscle tissue revealed myxospores with ‘*K. thyrsites*–like morphology’, characterized by stellate spores in shape with 4 unequal pyriform polar capsules (Fig. 2) (Gilchrist 1924; Whipps and Kent 2006; Heiniger et al. 2013; Giulietti et al. 2020). A total of 27 partial SSU rDNA sequences (1287 bp) were obtained from the liquefied muscle tissue of the sardines. These sequences were identical to each other and to those of *K. thyrsites* from sardine (OM200072-73) and Atlantic chub mackerel (*Scomber colias*) (MT991409) from the Iberian Atlantic, sardine from the Moroccan Atlantic (OR647554), silver scabbardfish (*Lepidopus caudatus*) from the Mediterranean Sea (MT912847), and Atlantic mackerel from the southern Norwegian Sea (MT913636-7) and the North Sea off southern England (AY542482).

**Table 1:** Sample size, fish host biometrics, and occurrence of *Kudoa*-induced ‘soft flesh’ occurrence in Iberian sardines from off the northern Spanish Atlantic coast (Cantabrian Sea) (ICES division 8.c.). *TL*, total length (mm); *TW*, total weight (g); *SD*, standard deviation

	Biometrics—all fish examined
N fish examined	500
N fish measured	417
Sex distribution f/m	248/169
TL	188 ± 21 (146–240)
TW	53 ± 19 (21–106)
Fulton’s K	0.8 ± 0.1 (0.6–0.9)
	**Biometrics—‘soft flesh’ affected fish**
N ‘soft flesh’ affected fish	27
Occurrence ‘soft flesh’ (%)	5.4%
TL	198 ± 22 (150–240)
TW	63 ± 21 (26–99)
Fulton’s K	0.8 ± 0.1 (0.6–0.9)
	**Biometrics—intact fish**
TL	188 ± 21 (146–238)
TW	52 ± 19 (21–106)
Fulton’s K	0.8 ± 0.1 (0.6–0.9)

There was no significant difference in the occurrence of ‘soft flesh’ between male and female sardines (Fisher’s exact test, *p* > 0.05). However, sardines showing ‘soft flesh’ were significantly larger (*n* = 27; mean TL ± SD: 198 mm ± 22) compared to those without the condition (*n* = 390; mean TL ± SD: 188 mm ± 21) (Mann–Whitney *U* test, *p* < 0.05). Fulton’s K did not differ between sexes. Mean length at age values estimated from the ALK were 187 mm for sardines aged ≤ 2 years, and 199 mm for those aged ≥ 3 years.

## Discussion

### Occurrence of ‘soft flesh’ in Iberian sardine stock

Despite the economic importance of the Iberian sardine stock, knowledge on *Kudoa*-induced ‘soft flesh’ in this stock remains limited. This study is the first to provide quantitative data on the occurrence of *Kudoa*-induced ‘soft flesh’ in the Iberian sardine stock from the northern Spanish Atlantic coast (ICES division 8.c), addressing a critical research gap in the literature. Our findings, which confirm *K. thyrsites* as the causative species through molecular identification, offer novel insights into the occurrence of this condition in Iberian sardines and related economic and management implications.

Knowledge on the occurrence of ‘soft flesh’ in sardines is mostly limited to the northern African stock (34.1 zone A + B) (Giulietti et al. 2024), with only fragmented data available for other sardine stocks (Menezes et al. 1990; Gilman and Eiras 1998; Cruz et al. 2011; Iglesias et al. 2022). Previous studies on *Kudoa* infection in the Iberian sardine stock, primarily based on market samples from Portugal, often lacked comprehensive quantitative data on the occurrence of ‘soft flesh’ and did not consistently employ standardized procedures for its assessment. Molecular identification of the causative parasite species was frequently absent, increasing the risk of misidentification. This is particularly important considering the co-occurrence of morphologically similar ‘soft flesh’–inducing species, such as *K. thyrsites* and *K. encrasicoli*, in Iberian Atlantic waters, with the latter exhibiting spore morphology that resembles that of *K. thyrsites* (Giulietti et al. 2024; Iglesias et al. 2022). Previous reports from Portugal by Menezes et al. (1990) and Gilman and Eiras (1998) documented *K. thyrsites*–like spores in sardines with flesh softening, but without providing molecular identification and quantitative data on the occurrence of ‘soft flesh’. Cruz et al. (2011) documented ‘soft flesh’ in 30% (27/90) of sardines from a fish market in the northern Portugal, but again without molecular confirmation of the causative species. Iglesias et al. (2022) also reported *K. thyrsites* infections in sardines from the Iberian stock off northern Portugal and the northwest coast of Spain (Division 9.a.CN and 9.a.N, respectively), but did not provide data on the occurrence of ‘soft flesh’. These inconsistencies between studies contribute to uncertainty regarding the occurrence of ‘soft flesh’ in the region and hamper comparisons.

To address these issues, this study employed standardized procedures, including controlled fish storage condition, preliminary microscopic analysis of the suspected infected fish, and molecular confirmation of the infection and causative species, detailed by Giulietti et al. (2022a, b, 2024) and Levsen et al. (2008). The occurrence of *K. thyrsites*–induced ‘soft flesh’ was 5.4% in the sampled population of the Iberian sardine stock. This rate is higher than the 1.4% (7/500) found by Giulietti et al. (2024) in the northern African sardine stock from off Atlantic Morocco (34.1 zone A). The methodologies used in both studies were consistent, enabling a direct comparison of ‘soft flesh’ occurrence data between these different stocks. Sardines from the northern African stock (34.1A + B) (TL 181 mm ± 16) (Giulietti et al. 2024) and the Iberian stock (TL 188 mm ± 21) were similar in size, thus ruling out a possible effect of fish size on *K. thyrsites*–induced ‘soft flesh’. Instead, the differences between these stocks may reflect variations in *K. thyrsites* burden, possibly influenced by the life history of the sardine stocks and the ecological characteristics of their respective ecosystems, particularly the distribution and occurrence of the parasite in susceptible host(s), including the putative definitive invertebrate hosts which to date remain unknown (Eszterbauer et al. 2015).

Our results also revealed that ‘soft flesh’–affected sardines were significantly larger than unaffected ones. This finding aligns with previous studies on European sardines, Atlantic mackerel, and Atlantic salmon (*Salmo salar*), showing a higher susceptibility to ‘soft flesh’ in larger fish due to higher prevalence and density of *K. thyrsites* (Gilman and Eiras 1998; Levsen et al. 2008; Jones and Long 2019; Giulietti et al. 2022a). Older fish, exposed to the infective stage of the parasite for longer periods, tend to accumulate more parasites, increasing the likelihood to develop ‘soft flesh’ (Castro et al. 2018; Casal et al. 2019). Moreover, a larger body size increases the surface area available for the infective stage of *Kudoa* spp. (Levsen et al. 2008; Jones and Long 2019; Giulietti et al. 2022a). According to the age-length key from the PELACUS0423 survey, the sardines affected by ‘soft flesh’ had a mean length of 198 mm and were estimated to be age 3 or older, while unaffected sardines had a mean length of 188 mm and were estimated to be age 2 or younger (unpublished results). These findings support the hypothesis that older fish, due to prolonged parasite exposure, are more likely to develop ‘soft flesh’ (Levsen et al. 2008; Castro et al. 2018; Casal et al. 2019; Giulietti et al. 2022a). Furthermore, no relationship was found between the sex of the sardines and the development of ‘soft flesh’, which is consistent with other studies in sardines and other small pelagic fish species (Gilman and Eiras 1998; Giulietti et al. 2022a).

### Economic and management implications

EU regulations require that seafood businesses perform visual inspection of fishery products to prevent parasitized ones from entering the market (EC 2002, 2004). However, the regulation does not address *Kudoa* parasites, as they are usually not associated with human health issues (EFSA BIOHAZ Panel 2024). Nevertheless, the unsightly appearance of ‘soft flesh’–affected fish fillets represents a food quality issue (Levsen et al. 2008). Detecting *Kudoa* infections in fresh fish is challenging for the quality control personnel, as infected fish may exhibit ‘soft flesh’ between 6 and 48 h after fish death (Giulietti et al. 2022a). Fish products that do not conform to quality standards are often discarded at various stages in the supply chain, or by consumers, undermining consumer confidence and causing financial losses to the fisheries sector (reviewed by Levsen 2015, and by Bolin et al. 2021). For instance, *K. thyrsites* infections have been associated with up to 4% annual production loss in Norwegian Atlantic mackerel (Giulietti et al. 2022a). In the Iberian sardine stock, the presently documented *K. thyrsites*–induced ‘soft flesh’ significantly degrades the quality and the marketability of the fish products, potentially leading to food spoilage and diminishing consumer confidence. This is particularly concerning for Spain, where sardines have substantial economic and cultural importance, with an annual consumption per capita of approximately 0.5 kg (Vázquez-Rowe et al. 2014; Molina-Fernández et al. 2015; Statistica 2023). On a wider scale, the ‘soft flesh’ issue could impact Spanish seafood’s reputation in key export markets, potentially reducing global demand. As one of Europe’s largest sardine suppliers, Spain may face economic and reputational consequences, especially since larger sardines, which are most affected, hold high value in both domestic and international markets (Silva et al. 2015).

The current lack of knowledge on the life cycle and transmission pathway of *Kudoa* species limits management strategies to prevent *Kudoa* infections in fish. Given the economic and cultural importance of the Iberian sardine stock, monitoring programs should be established to investigate the potential risk factors associated with *Kudoa* infections.

## Conclusions

The present study represents the first investigation of occurrence of *Kudoa*-induced ‘soft flesh’ in the Iberian sardine stock and identified the causative species *Kudoa thyrsites* using molecular methods. The findings indicate 5.4% occurrence of ‘soft flesh’, with larger and older sardines being the most affected. This research addresses gaps in the literature, as previous studies lacked consistent methodologies for assessing the occurrence of ‘soft flesh’ and confirming the parasite species, thus limiting the understanding of this condition and its economic implications in such an important sardine stock. *Kudoa* infections pose economic risks, affecting the marketability of sardines and leading to loss of consumer confidence in both domestic and international markets.

## Data Availability

No datasets were generated or analysed during the current study.
